# Dynamic Periocular Wrinkle Patterns: An Anatomical Study on Young Adults

**DOI:** 10.1111/jocd.70215

**Published:** 2025-04-30

**Authors:** Istemihan Coban, Fulya Yaprak Erkmen, Gülsüm Derya Aktaş

**Affiliations:** ^1^ Department of Anatomy, Faculty of Medicine Izmir Democracy University Izmir Turkey

**Keywords:** anatomy, eyelids, facial expression, facial muscles, skin aging, wrinkles

## Abstract

**Background:**

Aging causes facial wrinkles, especially dynamic wrinkles related to mimic movements, predisposing areas to static wrinkles from a young age, notably in the periorbital region. This study aims to analyze dynamic wrinkle patterns on periorbital skin during mimic movements in healthy young adults to identify wrinkle precursor regions.

**Methods:**

Dynamic periorbital wrinkle patterns were analyzed in 184 healthy adults (93 females, 91 males) aged 18–24 years. Standardized facial photographs were taken at rest and during various mimic movements. The periorbital region was divided into superior, inferior, lateral, and medial subregions; wrinkle patterns were classified and analyzed. Interobserver and intraobserver reliability were assessed.

**Results:**

In the upper periorbital region, the most common wrinkle pattern was oblique lines extending from the medial and lateral canthus to the upper corners (Type 3, 56%). Inferiorly, the most common pattern was a linear wave from the lateral canthus to the lateral margin (Type 3, 25.5%). Medially, no specific pattern (Type 6, 33.2%) and an arrowhead‐like motif directed toward the medial canthus (Type 2, 20.7%) were prevalent. Laterally, radial waves extending from the lateral canthus to the lateral brow tip were most common (Type 1, 34.8%). The absence of a distinct wrinkle pattern in the medial region was more common in men than in women (48.4% vs. 17.6%). Males were more likely to have wrinkle patterns consisting of oblique short lines in the inferolateral direction in the inferior region (Type 2, 31.2%). Miniature crow's feet‐like wrinkles were more common in females.

**Conclusion:**

This study presents a new classification of dynamic wrinkle patterns in the periorbital region in young adults, highlighting sex differences.

## 1 Introduction

Aging is a complex process that occurs under the influence of intrinsic and extrinsic factors. During the process, all tissues, epithelial, connective, muscular, and nervous, undergo changes to varying degrees between individuals, while individuals with a similar chronologic history may present with different findings. Aging findings that occur in the facial region, which is critical for human beings, a biopsychosociocultural creature, to maintain their ordinary social lives, are perhaps placed in a separate place compared to other parts of the body that can be visually examined. The aging process in the facial region may have a greater impact on self‐esteem and interpersonal relationships. There is an increase in the number and variety of rejuvenative procedures performed to reduce, make less visible, and even delay the effects of aging in the facial area [1]. From an anatomical point of view, the facial region, which has multi‐layered layers, is quite complex. Topographically, the anatomical features of the skin, fascial cover, fat clusters, mimic muscles, vascular‐nerve branches, ligaments, and bones and the changes that occur during the aging process vary regionally [2]. Changes in all these structures due to intrinsic and extrinsic factors produce visible results, especially on the skin of the face. Foldings and wrinkles, which are one of the first and most striking signs of the aging process, are very characteristic of the face, especially those in the periorbital region. Wrinkles can be categorized into two main types: the static type, which can be recognized in a neutral‐resting state, and dynamic wrinkles that become evident during facial expressive movements. Intrinsic (ethnicity, anatomical variations, hormonal changes) and extrinsic (lifestyle influence, smoking and nicotine, exposure to UV light) factors play an important role in the formation of these two types of wrinkles. In terms of anatomical variations counted among intrinsic factors, not only skin thickness or fat composition but also the direction of extension and the starting and ending points of the muscle fibers that produce mimic movements, the specific attachment and placement of the suspensory ligaments that connect the muscle to the deeper layers or periosteum, and changes in the elevating effect of the adipose tissue clusters between the layers with the aging process (such as atrophy of the deep ones and increase in the surface ones) are the basic anatomical factors [3, 4]. There are studies in the literature that reveal the degree of dynamic and static wrinkles in the periocular region, which is one of the regions of the midface most affected by dynamic wrinkles [5, 6, 7]. but there are few studies that specifically examine the dynamic fold patterns in the periocular region [8, 9, 10]. The facial expression movements that the person makes from the moment he/she learns to make mimic movements become visible as fold lines (dynamic wrinkles) in the skin tissue in the chronic period. In the following process, dynamic wrinkle lines can be detected as facilitating areas for the formation of static wrinkle lines. Anti‐aging treatments can be tailored to each person by finding these precursor fold lines [4]. Therefore, understanding the relationship between the pattern of dynamic wrinkling and future static wrinkling before the static wrinkle is visible may be useful for determining early intervention protocols. Given the lesser impact of aging processes such as epidermal thickening, decreased skin elasticity, atrophy of fat compartments, and loss of facial bone volume, analysis of periocular skin wrinkle type in young adults may provide important information for individualized analysis of precursor areas of aging for individuals by better understanding the fold areas associated with chronic muscle movement [11]. The aim of this study was to reveal wrinkle precursor regions by analyzing the dynamic fold patterns that occur in the periocular skin with various mimic movements in healthy young adults without any history of cosmetic or surgical procedures, facial injuries, or chronic diseases.

## 2 Materials and Methods

Dynamic periocular wrinkle pattern analyses were conducted on a group of healthy young adults (93 females and 91 males) aged 18–24 years, all of whom were free from chronic diseases, had no history of facial conditions or injuries, had never undergone cosmetic procedures such as botulinum toxin or filler treatments, and were non‐smokers. Participants were randomly selected as volunteers for this cross‐sectional study, and their age, weight, and height were recorded.

Photographs of the facial region of the volunteers were taken with a digital camera (Nikon D3300) in the frontal and sagittal planes by providing a suitable bright environment and photographing the head region in the neutral anatomical position first in the resting state (without mimic movement) and then in the same order by asking the person to make various mimic movements in the strongest state that the person can do. The photographs were analyzed with the ImageJ software [12].

The periocular lateral region is defined as the area between the line extending from the lateral canthus to the lateral end of the eyebrow and the line from the lateral canthus to the zygion. The medial periocular region lies between the line connecting the medial canthus to the nasion and the line from the medial canthus to the rhinion. The inferior periocular region is located between the medial and lateral periocular regions, just below the palpebral fissure. The periocular upper region is the area above the palpebral fissure, bounded superiorly by the lower border of the eyebrow.

To provoke dynamic wrinkles, the volunteers were first photographed at rest, and then asked to perform various facial expressions. These included raising the eyebrows, frowning, wrinkling the nose, lifting the upper lip, smiling (lifting the lip commissures without showing the teeth), adopting a dissatisfied expression (pulling the lip commissures downward with the mouth naturally closed), pressing the lips together (as if holding a piece of paper between the lips), and stretching the lips (pulling the lip commissures to the sides) with the chin in full occlusion in addition to baring teeth (separating the upper and lower lips as far as possible while the teeth are closed), vocalizing the letters “O” and “U,” closing the eyes in a natural manner, squinting, and forcefully closing the eyes. Sufficient rest was given in a neutral position between each movement. Photographs were taken from the opposite, 45° right and left angles of the individuals at the time of the movements, with appropriate room lighting and at a distance of 60 cm.

In the study inter‐ and intra‐observer reliability analyses of dynamic skin wrinkle patterns in the periocular region were performed independently assessed by three anatomists. The observers' examinations were repeated on different days and the reliability and consistency of the classifications obtained were analyzed. Interobserver agreement was analyzed using the Fleiss' Kappa coefficient. As a result of the analysis, the average Fleiss' Kappa value was calculated as 0.83 for all regions. This result indicates an “excellent” level of agreement between the observers. Among the periocular regions, the highest agreement was obtained in the lateral periocular region evaluations (Kappa = 0.87) and the lowest agreement was obtained in the lower periocular region evaluations (Kappa = 0.77). However, both values were above 0.75, indicating that the overall agreement was quite good. Intra‐observer reliability was calculated using the Intraclass Correlation Coefficient (ICC) to assess the consistency within the observers. The mean ICC value for the consistency of the evaluations made by each anatomist on different days was 0.89. This result indicates that intra‐observer reliability was “excellent”.

In the study, the periocular region was divided into four main regions (superior, inferior, lateral and medial) and the wrinkle types observed in each region were classified separately. Frequency and percentages of the types were determined for each region. Multinomial logistic regression analysis was used to compare the distribution of wrinkle types between genders. The statistical significance level was accepted as *p* < 0.05. All statistical analyses were performed using SPSS 26.0 software.

## 3 Results

### 3.1 Participant Demographics

The study group consisted of 93 healthy women and 91 healthy men aged 18–24 years with a mean age of 20.5 ± 1.4 years. The mean height of the case group was 172.7 ± 9.1 cm (range: 155–200 cm), mean weight was 68.3 ± 15.8 kg (range: 39–125 kg), and mean body mass index (BMI) was 22.7 ± 3.9 kg/m^2^ (range: 14.9–40.3 kg/m^2^). The mean age of male subjects was 20.8 ± 1.5 years, mean height was 179.1 ± 7.2 cm (range: 160–200 cm), mean weight was 77.9 ± 14.7 kg (range: 40–125 kg) and mean BMI was 24.2 ± 4 kg/m^2^ (range: 15.6–40.4 kg/m^2^). The mean age of female subjects was 20.1 ± 1.2 years, mean height was 166 ± 5.2 kg/m^2^ (range: 155–180 kg/m^2^), mean weight was 58.5 ± 9.6 kg, and the mean BMI was 21.2 ± 3.3 kg/m^2^ (range: 14.9–34.3 kg/m^2^).

### 3.2 Overall Wrinkle Patterns

**FIGURE 1:**
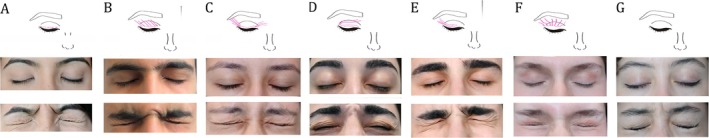
Dynamic wrinkle patterns in the upper part of the periocular region. The patterns of wrinkles induced by natural facial posture, gentle eye closure, and forceful eye closure are presented. During gentle eye closure, no distinct features are observed in the periorbital region; however, significant differences in wrinkle patterns become evident with forceful eye closure. (A) Type 1, a flat wrinkle line along the lower margin of the upper tarsal cartilage; no dynamic wrinkles extending toward the brow line are observed. (B) Type 2, a wave‐like pattern of wrinkles extends from the upper tarsal cartilage line toward the superolateral direction. (C) Type 3, a ribbon‐like wrinkle pattern extends from the upper tarsal cartilage to both the superomedial and superolateral regions. (D) Type 4, parallel wrinkles form a pseudo‐dermatochalasis, giving the appearance of a skin pile on the upper eyelid. (E) Type 5, unlike Type 2, a lateral accumulation of wrinkles is observed. (F) The vertical radial wave pattern emanating from the upper eyelash line in all directions is particularly notable. (G) Type 7, transverse lines accumulate between the upper tarsal cartilage and the eyelashes; however, no distinct wrinkle pattern is discernible on the upper eyelid.

**FIGURE 2:**
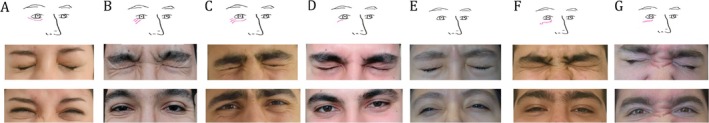
Dynamic wrinkle patterns in the inferior part of the periocular region. Each series begins with displaying wrinkle patterns in the periocular region induced by tight eye closure, and the second image shows wrinkle patterns in the periocular region prompted by teeth‐showing or smiling expressions (at the bottom). (A) Type 1, a broad wrinkle originates from the medial aspect of the infraorbital margin and extends horizontally toward the lateral side. (B) Type 2, peripheral wrinkle lines appearing during tight eye closure become most prominent with a smile. (C) Type 3, short wrinkle lines are observed between the main inferolateral wrinkle line and the lateral canthus. (D) Type 4 resembles a single corner line appearance located lateral to the midpupillary vertical line. (E) Type 5 is not induced by movement, is well‐supported topographically by the underlying connective tissue, and does not form a distinct wrinkle pattern. (F) Type 6 exhibits a pattern extending from the medial canthus to the lateral canthus, with curls observed on the lateral half. (G) Type 7, an unipennate wrinkle line is visible.

**FIGURE 3:**
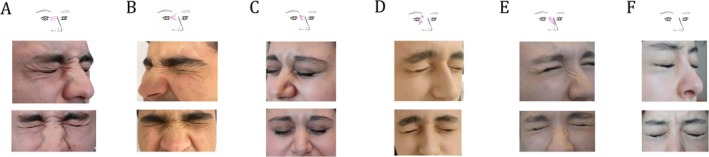
Dynamic wrinkle patterns in the medial part of the periocular region. The wrinkle types in this area, which become more prominent with tight eye closure, are observed from lateral and anterior angles. (A) Type 1, wrinkle lines extending toward the nasion form a zigzag pattern when viewed from the front. (B) Type 2 consists of wrinkle lines originating from the nasion and rhinion lines that converge at the medial canthus, forming an arrowhead‐like shape when viewed from the front. (C) Type 3, an oblique line extends toward the rhinion with less pronounced transverse lines above it. (D) Type 4, wrinkle lines radiate from the medial canthus, similar to beams of light, though they are shorter. (E) Type 5, wrinkle lines generally extend toward the rhinion, often in two distinct folds. (F) Type 6, no specific wrinkle pattern is detected, though a limited number of wrinkles are present along the medial canthus.

**FIGURE 4:**
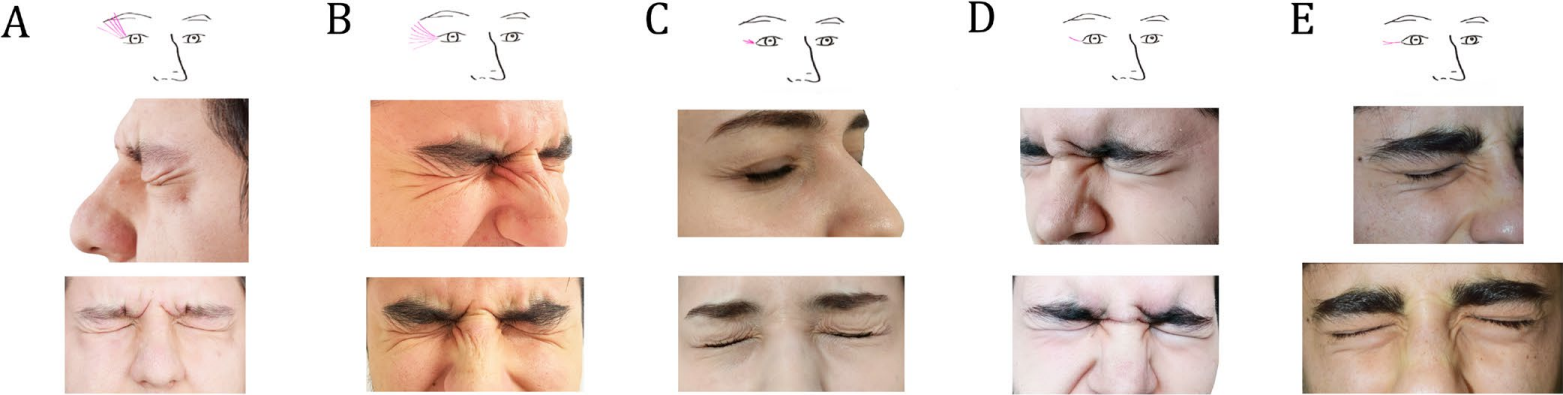
Dynamic Wrinkle Patterns in the Lateral Part of the Periocular Region. Each figure presents a wrinkle motif in the periocular lateral region induced by tight eye closure in the same individual. (A) Type 1, superolaterally directed oblique wrinkle lines above the level of the lateral canthus. (B) Type 2, wrinkle lines radiating radially both superiorly and inferiorly. (C) Type 3, a miniature crow's feet pattern. (D) Type 4, a wrinkle line dividing the upper and lower halves at the level of the lateral canthus. (E) Short radial wrinkle lines located above and below the lateral canthus, representing an early form of Type 3.

Seven different pattern types were detected in the upper part of the periocular area (Figure 1). Among these, the most common type is the oblique fold line (Type 3, 56%) extending from the medial canthus and lateral canthus to the upper medial and lateral corners of the region. The second most common type is the wrinkle extending from the lateral canthus to the lateral corner of the periocular area (Type 5, 19%). In the inferior part of the periocular area, seven different fold patterns have been defined, of which the most common type is Type 3 (25.5%), which is a linear wave motif that is bounded by the lateral canthus and the lateral edge of the area, and the second most common type is Type 2 (21.7%), which has oblique parallel lines inferolaterally between the lateral canthus and the midpupillary line. The third most common pattern is Type 6 (20.1%) with transverse‐oblique extension starting from the medial canthus and extending to the lateral canthus (Figure 2). In the medial part of the periocular region, there are Type 6 (33%), which does not follow a specific pattern, Type 2 (20.6%), which forms an arrowhead‐like motif pointing to the medial canthus, and Type 3 (12%), which appears as an oblique‐vertical line extending from superior to inferior, medial to lateral, and short transverse lines emerging from it (Figure 3). In the lateral region of the periorbita, five types have been defined. Among these, the most common types are Type 1 (35%), which expresses radial waves with vertical‐oblique extension from the lateral canthus to the lateral end of the eyebrow, concentrated in the upper lateral corner, Type 2 (30%), which contains folds extending from the lateral canthus to the upper and lower corners but with a more transverse course, and miniature crow's feet pattern Type 3 (20%), which contains short curved waves emerging from the lateral canthus (Figure 4).

### 3.3 Statistical Findings

In 4% of females and 2% of males, no specific fold pattern was detected in the periocular upper area, in 18.6% of females and 8.6% of males in the periocular lower area, and in 17.2% of females and 48.3% of males in the periocular medial area. The distribution rates of dynamic wrinkles provoked by facial expression in the periocular region are given in Tables 1, 2, 3, 4. A dynamic fold pattern occurs in the periocular lateral area in all cases. Multinomial logistic regression analysis was performed to evaluate the relationship between periocular fold types (superior, inferior, lateral and medial) and gender. The results of the analysis for each region are summarized in Table 5 below.

**TABLE 1:** Distribution of dynamic fold patterns in the upper part of the periocular region according to gender.

Dynamic wrinkle formation	Males (*N* = 93) (%)	Females (*N* = 91) (%)	Total (*N* = 184) (%)
*Type 1* (transverse wrinkles only in the upper tarsal cartilage line)	4 (4.3%)	17 (19%)	21 (11.4%)
*Type 2* (wrinkles starting over the upper tarsal cartilage and extending superolaterally)	2 (2.2%)	9 (9.9%)	11 (6%)
*Type 3* (oblique wrinkles extending from the medial canthus and lateral canthus line to the upper medial and lateral corners of the region)	59 (63.4%)	44 (48%)	103 (56%)
*Type 4* (wrinkles in the form of two separate horizontal bands on the tarsus and on the superior palpebra)	3 (3.2%)	1 (1.1%)	4 (2.2%)
*Type 5* (wrinkles extending from the lateral canthus to the lateral corner of the orbit)	22 (24%)	13 (14.3%)	35 (19%)
*Type 6* (wrinkles in the form of radial beams from the upper tarsal cartilage to the superior)	1 (1.1%)	3 (3.3%)	4 (2.2%)
*Type 7* (no discernible wrinkle form)	2 (2.2%)	4 (4.4%)	6 (3.3%)

**TABLE 2:** Distribution of dynamic fold patterns in the inferior part of the periocular region according to gender.

Dynamic wrinkle formation	Males (*N* = 93) (%)	Females (*N* = 91) (%)	Total (*N* = 184) (%)
*Type 1* (transverse from the inferior tarsal cartilage to the lateral canthus line, mostly as a single continuous line, rarely interrupted)	4 (4.3%)	20 (22%)	24 (13%)
*Type 2* (inferolaterally directed oblique short lines from the inferior tarsal cartilage to the lateral canthus)	29 (31.2%)	11 (12.1%)	40 (21.7%)
*Type 3* (a single line extending laterally from the inferior tarsal cartilage and several small fold lines above the line toward the lateral canthus)	31 (33.3%)	16 (17.6%)	47 (25.5%)
*Type 4* (a single oblique wrinkle line starting from the line passing through the vertical midpupillary line and directed inferolaterally)	4 (4.3%)	1 (1.1%)	5 (2.7%)
*Type 5* (no discernible wrinkle form)	8 (8.6%)	17 (18.7%)	25 (13.6%)
*Type 6* (wrinkle line extending between from the medial canthus to the lateral canthus and accompanied by curls at superolateral part of the lateral end)	14 (15.1%)	23 (25.3%)	37 (20.1%)
*Type 7* (wrinkle line extending between the medial canthus and the lateral canthus and accompanied by bifurcations at the end)	3 (3.2%)	3 (3.3%)	6 (3.3%)

**TABLE 3:** Distribution of dynamic fold patterns in the medial part of the periocular region according to gender.

Dynamic wrinkle formation	Males (*N* = 93) (%)	Females (*N* = 91) (%)	Total (*N* = 184) (%)
*Type 1* (horizontal‐oblique wrinkles extending from the medial canthus to the nasal dorsum‐nasion line)	0 (0%)	8 (8.8%)	8 (4.3%)22
*Type 2* (wrinkles coming from the nasion and rhinion line and merging at the medial canthus, forming an arrowhead‐like appearance)	22 (23.7%)	16 (17.6%)	38 (20.7%)
*Type 3* (a long oblique wrinkle perpendicular to the medial canthus line and short wrinkles directed laterally from this line to the area between the nasion and rhinion)	4 (4.3%)	18 (19.8%)	22 (12%)
*Type 4* (small vertical wrinkles extending perpendicular to this line above and below the medial canthus line)	6 (6.4%)	1 (1%)	7 (3.8%)
*Type 5* (oblique wavy wrinkles oriented either to the nasion or to the rhinion)	21 (22.6%)	27 (30%)	48 (26.1%)
*Type 6* (no discernible wrinkle form)	45 (48.4%)	16 (17.6%)	61 (33.2%)

**TABLE 4:** Distribution of dynamic fold patterns in the lateral part of the periocular region according to gender.

Dynamic wrinkle formation	Males (*N* = 93) (%)	Females (*N* = 91) (%)	Total (*N* = 184) (%)
*Type 1* (wrinkle lines on the lateral canthus line extending toward the lateral end of the eyebrow)	35 (37.6%)	29 (31.9%)	64 (34.8%)
*Type 2* (horizontally oriented wrinkle waves radiating from the lateral canthus line)	34 (36.6%)	21 (23%)	55 (29.9%)
*Type 3* (miniature crow's feet‐like appearance starting from the lateral canthus line and limited to peripheral distribution)	8 (8.6%)	29 (31.9%)	37 (20.1%)
*Type 4* (a short transverse wrinkle line on the lateral canthus forming a horizontal separation between the upper and lower eyelid)	6 (6.5%)	1 (1.1%)	7 (3.8%)
*Type 5* (a short transverse wrinkle line starting from the lateral canthus, resembling a left‐slanted letter Y, bifurcated peripherally)	10 (10.8%)	7 (7.7%)	17 (9.2%)

**TABLE 5:** Results of statistical analysis that show the relationship between periocular wrinkle types and gender.

Periocular region	Chi‐square	*p*	Nagelkerke R2	Significant effect of the sex	Key findings
Superior	20.764	0.192	0.115	No	Males more likely to have dynamic wrinkles from lateral canthus to lateral corner
Inferior	40.731	< 0.001	0.208	Yes	Males 92.1% less likely to have small transverse lines from oblique long line
Lateral	16.926	0.010	0.093	Yes	Lateral crow's feet significantly less frequent in males (*p* = 0.010)
Medial	40.731	0.002	0.208	Yes	Males 72.3% less likely to have transverse waves to nasion (*p* = 0.002)

## 4 Discussion

The facial regions where dynamic wrinkle patterns are most clearly distinguishable are the glabellar and periocular regions [13]. Dynamic and static wrinkle features are of interest to researchers in computer science, forensic science, and medical sciences for face analysis and facial recognition, age and gender determination [5, 14, 15]. Although criteria such as the Lemperle Wrinkle Assessment Scale, modified Fitzpatrick Wrinkle Scale, or Clinician's Global Aesthetic Improvement Scale are frequently used to evaluate the visible signs of the static wrinkles, there is no commonly accepted classification or terminology for the anatomical or kinetic evaluation of dynamic wrinkles [5, 16, 17]. Whether it is to understand the individual characteristics of facial appearance, to determine the visible effects of aging that are specific to individuals, or to monitor the success of anti‐aging treatments, it is necessary to determine not only the difference between the indentations and protrusions of the skin layers and wrinkle severity, but also the anatomical location and extension lines of facial wrinkle patterns, especially dynamic wrinkle patterns. By understanding the dynamic wrinkle pattern, anatomical differences in the mechanism of wrinkle formation can be better understood and individualized treatment options can be determined. Understanding the dynamic wrinkle‐based anatomical status that occurs in the facial region of individuals in their youth, when the effects of aging are not seen, is a prerequisite for understanding the aging process in the face, because an uncountable number of fold areas are formed in certain sub‐regions from childhood when mimic movements begin to be made, and after a certain age, dynamic wrinkles begin to settle in some places with the stagnation of anatomical formations after puberty and the settlement of mimic movement patterns. In support of this view, it has been shown that the severity of dynamic folds on the face increases with age and the distribution patterns of dynamic and static wrinkles are similar [4, 18]. In these repetitive folding areas, mechanical structural changes occur in the architecture of the soft tissue and skin texture, along with localized differences in the regions affected by sunlight [19, 20].

The results of the present study add valuable information to the literature as it is one of the first studies to describe periocular dynamic fold patterns in the skin of four different subregions surrounding the eye. There are very few studies on the dynamic skin folds in the upper area between the eyebrows and palpebral fissure. One of the most frequently emphasized issues is upper eyelid sagging that may limit the visual field, cause eye irritation, lead to headaches, and create a cosmetically aged appearance [21, 22, 23]. Although genetic factors are known to be an important factor in upper eyelid sagging, gender, light skin color, high BMI, and smoking are also factors that increase the risk of sagging, and reports indicate that 16% of individuals aged 45 years and older have sagging eyelids, with 19% of men and 14% of women affected [21]. It was observed that the dynamic lines formed in the upper eyelid skin during closing or squinting movements generally extend between the upper tarsal cartilage and the eyebrow line, similar to an oblique‐radial network shape. Muscle fibers forming a right angle with these lines wrap the upper eyelid posteriorly in a bandage‐like manner. Generally, transverse lines in the skin covering the upper tarsus are prominent, whereas in some types, transverse dynamic wrinkles extending to the periphery have been observed. The most interesting of these is Type 5, and in cases with this pattern, the wrinkles tend to extend transversely from the exocantion to the lateral corner of the periocular area. Considering that the lines forming this wrinkle form a right angle, it can be predicted that this Type 5 dominant individual may be predisposed to upper eyelid sagging with the activity of the orbicularis oculi muscle in the chronic process. The fact that 19% of the cases exhibited this pattern further supports the previous reports. Interestingly, it was reported that damage to the orbicularis oculi muscle may impair the development of dermatochalasis [22].

The medial part of the periocular area is perhaps the least focused region in periocular dynamic wrinkle research. Dynamic wrinkle patterns around the medial palpebral ligament on the medial side of the periocular region were examined for the first time in our study, and patterns dominated by dynamic wrinkles extending from the nasofrontal junction and the nasal bone‐cartilage junction toward the medial canthus were identified. It has been observed that wrinkle patterns in the medial line occur with movements such as squinting, closing the eyes, smiling, as well as with movements such as frowning, wrinkling the dorsum of the nose, and showing teeth. Although wrinkles in the medial region appear to trigger transverse wrinkle lines in the nasal dorsal course in most cases, the number of cases in which no wrinkles were seen in the medial region in any of the young cases examined is not less (approximately one third of the cases). In these cases, the fibers of the depressor supercilii, procerus, levator labii alaque nasi, nasalis muscles did not participate in the movement of the lacrimal part of the orbicularis oculi either involuntarily or because of a learned movement pattern (no distinction could be made because electrophysiological monitoring of muscle contraction was not performed in the study) [24]. Facial muscles have complex interconnections that contribute to the intricacy of facial expressions. In the study of Hur et al. in which 44 cadavers were examined, the depressor supercilii muscle was directly connected with the levator labii superioris alaque nasi, or the inferior part of the orbicularis oculi, with a frequency of 75%, which supports the interpretation of the findings of our study in the medial region [25].

In the inferior area of the periorbita, the fold lines formed by the tight closure of the eyes around the lower eyelid and orbital rim were examined, but it is noteworthy that the nasojugal and palpebromalar grooves (static) were not evident in any of the cases. In addition to the orbicularis oculi, the effect of levator labii superioris, major and minor zygomatic muscles is also evident in the formation of dynamic folds in this area [26]. In the lower part of the periocular area, the wrinkles formed during squinting and closing of the eye are different. During squinting movements (also when smiling, showing teeth, wrinkling the dorsum of the nose, etc.), the soft tissue in the malar and infraorbital area is pulled superiorly, resulting in a dynamic wrinkle that corresponds to the line of the orbicularis retaining ligament, which defines the area between the infraorbital margin and the lower eyelid. This wrinkle was noted to be similar in shape between the cases. On the other hand, wrinkles provoked by eye closure movement show some differences between individuals and have been classified for the first time in this study. In the lower part of the periocular area, the wrinkles formed during squinting and closing of the eye are different. In the majority of cases, these lines were found to have a long wrinkle line extending from the medial canthal line laterally and from the top downwards. It is interesting to note that among these, the types extending from the long wrinkle line laterally in a vertical‐oblique direction (Type 2 and Type 3) were observed almost twice as often in males and may be related to the more severe lower eyelid sagging in males as reported in the study by Ezure et al. [27] In 13.5% of the current cases, no wrinkles were observed, indicating that the lower eyelid skin was not involved in the action during the eye closing.

The periocular lateral area is one of the most frequently treated and popular areas for cosmetic reasons (crow's feet and facelift). It is possible to find wrinkle pattern classification in the lateral region, which is not done in other periocular regions. Kane defined four types to distinguish dynamic wrinkles in this area of importance: Type I is the most common type, full or fan‐shaped wrinkles; Type II—lines extending to the lower eyelid/upper cheek line, are the second most common type; Type III—located only upper eyelid level; and Type IV wrinkles that located only around the lateral canthus, least common type [10]. Kane's work is only participatory and there is no specific age limit. Although the types' forms were similar in the young patients in our study, their frequencies were different from those Kane reported. In Kane's study, the prevalence was 47% for Type 1, 25% for Type 2, 18% for Type 3 and 10% for Type 4, while in our study it was 35% for Type 1, 30% for Type 2, 20% for Type 3, 6% for Type 4, and 9% for Type 5 [10]. Type 4 and Type 5 in our study can be considered subgroups of Type 4 described by Kane. The reason for subdividing this group into subgroups is that the bifurcation motif in the wrinkle line forms a more prominent antecedent pattern at the border of the lateral canthal ligament in this region, especially toward the upper eyelid level. The Type 5, seems to be the predecessor of the miniature crow's foot appearance classified as Type 3.

## 5 Limitations

In conclusion, while this study provides valuable insights into dynamic wrinkle patterns around the periocular region, several limitations should be noted. The lack of standardized classification for dynamic wrinkles and the absence of electrophysiological monitoring to directly assess muscle activity limit the precision and generalizability of the findings. The cross‐sectional design and lack of control for confounding variables, such as environmental and lifestyle factors, also introduce variability that may impact the results. Moreover, the study does not extensively explore the implications of these wrinkle patterns for cosmetic or therapeutic interventions, leaving room for further research to address these gaps and expand the understanding of dynamic wrinkles across different life stages and facial regions.

## 6 Conclusion

The study offers a novel perspective on the dynamic wrinkle patterns of the periocular region, specifically highlighting distinct patterns in four subregions around the eye. The identification and classification of these wrinkle patterns provide valuable anatomical and functional insights, contributing to a deeper understanding of facial dynamics and aging processes. The findings highlight the significance of researching wrinkle patterns beyond static evaluations, paving the way for more individualized approaches to cosmetic and therapeutic interventions, even in the absence of a generally recognized classification system for dynamic wrinkles. The study also reveals key gender‐related differences in wrinkle patterns, which may hold implications for tailored treatments in clinical and aesthetic practices. Future research, particularly longitudinal studies and those incorporating electrophysiological monitoring, will be essential to expand upon these findings, standardize wrinkle assessments, and explore the practical applications of dynamic wrinkle analysis in facial rejuvenation and anti‐aging therapies.

## Disclosure

Artificial Intelligence (AI) Usage Statement: AI‐assisted technologies were utilized exclusively as an aid in translation tasks for this article.

## Ethics Statement

This study was approved by Izmir Democracy University Non‐Interventional Procedures Local Ethics Committee on 21/07/2022 and numbered E‐35950415‐604.01.02‐26143.

## Consent

Approval of the Local Ethics Committee of Izmir Buca Seyfi Demirsoy Training and Research Hospital was obtained for the study (approval number: 2021/12–71). The study was completed in accordance with the Declaration of Helsinki. Due to the retrospective nature of our study, informed consent was not obtained for the study, but informed consent for inclusion in the scientific research is obtained from patients who are treated and followed up in the training and research hospital at the time of admission to the hospital. In addition, our study was approved and supervised by the hospital management and the hospital ethics committee.

## Conflicts of Interest

The authors declare no conflicts of interest.

## Data Availability

The data that support the findings of this study are available from the corresponding author upon reasonable request.
